# Theoritical Calculations in Structures and Functions of Proteins

**DOI:** 10.1063/4.0000892

**Published:** 2025-10-27

**Authors:** Yang Ha

**Affiliations:** Lawrence Berkeley National Lab, Berkeley, CA, USA

## Abstract

Theoritical calculations at quantum mechanic (QM) levels can provide additional insights than structures alone. For example, spin states can be extremely useful in metalloproteins; charge densities can be related to the function of proteins such as redox behavior or colors of fluorescence; quantum mechanic calculations can even model reactivities at the active site to couple with experimental kinetics data. In this presentation, I will show a few examples that with the help of theoretical calculations, the understanding of the structure and function of proteins can be greatly boosted.

